# Facile Synthesis of Ag NP Films via Evaporation-Induced Self-Assembly and the BA-Sensing Properties

**DOI:** 10.3390/foods12061285

**Published:** 2023-03-17

**Authors:** Jiahang Yu, Huixin Tian, Mingyuan Huang, Xinglian Xu

**Affiliations:** Key Laboratory of Meat Processing and Quality Control, Ministry of Education, National Center of Meat Quality and Safety Control, Jiangsu Collaborative Innovation Center of Meat Production and Processing, Quality and Safety Control, College of Food Science and Technology, Nanjing Agricultural University, Nanjing 210095, China

**Keywords:** Ag NP films, evaporation-induced self-assembly, BAs, meat detection

## Abstract

Herein, we design and prepare large-area silver nanoparticle (Ag NP) films based on evaporation-induced self-assembly, which offers the visual and real-time detection of chilled broiler meat freshness. The color change is based on the fact that an increase in the biogenic amine (BA) concentration causes a change in the absorption wavelength of Ag NPs caused by aggregation and etch of the Ag NPs, resulting in a yellow to brown color change, thus enabling a naked-eye readout of the BA exposure. The Ag NP films exhibit a rapid, sensitive, and linear response to BAs in a wide detection range of 2 µM to 100 µM. The Ag NP films are successfully applied as a quick-response, online, high-contrasting colorimetric sensor for visual detection of the freshness of chilled broiler meat.

## 1. Introduction

Modulating the localized surface plasmon resonance (LSPR) of noble metallic nanoparticles such as gold and silver have received widespread attention owing to their potential to offer the advantages of multifunctionality, less toxicity, large specific surface area, strong mechanical properties, and lower immune response. The bottom-up self-assembly of noble metal nanoparticles represents one of the needed tools in nanotechnology to build novel complex functional architectures, with the resulting assemblies showing corresponding superior collective nano properties over the individual nanoparticles for a wide range of applications [1,2]. Among different strategies, the use of surface and interfacial regulation to self-assemble noble metal nanoparticles has been used in a wide array of studies [3,4,5], which can be achieved typically by the magnetically assisted self-assembly [6], evaporation-induced self-assembly [7], electrostatically driven self-assembly [8], capillary-driven self-assembly [9], template-guided self-assembly [10], adsorption of the nanoparticles [11] and other methods [12,13].

In particular, evaporation-induced self-assembly has been shown to be a very effective and efficient method for directing the self-assembled nanostructures in the noble metal nanoparticles, which can find broad use in different applications such as catalysts [14], sensing [15], and medical fields [16]. Such a production of noble metal nanoparticles has many strengths because it is particularly suitable to material shaping at the microscopic scale (such as films, fibers, and thin microspheres) and allows the aggregation of many chemically incompatible components into confined selected shapes, thus opening up space for infinite potential chemical combinations, which could be impossible to carry out under thermodynamic control [17].

Recent research has focused on dynamic tuning of the LSPR properties of noble metal nanoparticles in a reversible and fast manner using external stimulus, such as magnetic field [18], light [19], temperature [20], and pH [21], which ultimately changes the local refractive index around them or the spacing between adjacent noble metal nanoparticles. However, this reversible plasmonic tuning of noble metal nanoparticles has been mainly implemented in liquid-phase conditions due to the lack of effective approaches in dynamically controlling noble metal nanoparticle spacing and surface charges in the solid state; this limitation restricts their potential in practical applications.

Live poultry markets are currently being restricted to reduce outbreaks of animal viruses. In this occasion, the consumption of low cholesterol, low-calorie, low-fat, and high-protein chilled broiler meat has grown significantly, which is the second largest consumed and traded meat in China. However, the production quality of chilled broiler meat determines its value [22]. Thus, the safety and freshness of chilled broiler meat have become a public health concern. The preset conditions limit such as improper handling, improper transport, and lack of cold storage facilities can easily lead to chilled broiler meat oxidation and spoilage, which often generates volatile amines, BAs, organic acids, sulfides, alcohols, aldehydes, and ketones, which have unpleasant and unacceptable off-flavors [23]. Among them, BAs are often used as a characteristic marker for detecting chilled broiler meat spoilage [24,25,26].

In this paper, a simple method for the preparation of large-area Ag NP films based on evaporation-induced self-assembly is presented, which is widely regarded as a typical evaporation-driven self-organization and self-assembly. This paper presents synthesis of Ag NP films with tunable plasma properties by limited-ligand-protection strategy. The presence of polyvinyl alcohol (PVA) and glucose ligands on a Ag NP surface allows dynamic color change and particle assembly through manipulation of Ag NP surface charges by exposure to exogenous substances. The mixed solution was prepared by mixing PVA, glucose, and silver nitrate (Ag NO_3_), which were oven-dried for approximately 5 h at 50 °C to evaporate the water, resulting in the formation of large-scale self-assembled Ag NP films. Moreover, the Ag NP films can be used as substrates for the detection of BAs. Further, the Ag NP films can be used to indicate the freshness of vacuum-packaged chilled broiler meat.

## 2. Materials and Methods

### 2.1. Materials and Reagents

PVA 1750, glucose, and silver nitrate (AgNO_3_) were purchased from Shanghai Alading Co., Ltd., (Shanghai, China). L-2-chlorophenylalanine was purchased from Shanghai Hengbai Biotech Co., Ltd. (Shanghai, China), and all the other BAs were purchased from Shanghai Yuanye Bio-Technology Co., Ltd. (Shanghai, China). Chilled broiler meat was purchased from Jiangsu Lihua Animal Husbandry Co., Ltd. (Jiangsu, China). Tyvek paper was purchased from Beijing 3M Co., Ltd. (Beijing, China).

### 2.2. Synthesis of Ag NP Films

After the addition of ultrapure water (99 mL) and PVA 1750 (3 g), the mixture was stirred for 0.5 h at 80 °C until completely dissolved; subsequently AgNO_3_ solution (1 × 10^−1^ M, 1 mL) and glucose (0.2 g) were added. The mixture was then stirred for 10 s at 80 °C. The mixed solution was oven-dried for approximately 5 h at 50 °C until yellow Ag NP films were generated.

### 2.3. Synthesis of PVA–Glucose Films

PVA 1750 (3 g) was added to ultrapure water (100 mL) and stirred for 30 min at 80 °C until completely dissolved. This was followed by the addition of glucose (0.2 g). The mixture was then stirred for 10 s at 80 °C. The 10 mL of mixed solution was transferred to 8-centimeter-diameter round Petri dishes and oven-dried for approximately 5 h at 50 °C until transparent and colorless film was generated.

### 2.4. Detection of Total Viable Counts (TVCs)

This method referred to our previous work [27].

### 2.5. Detection of Total Volatile Basic Nitrogen (TVB-N)

This method referred to our previous work [28].

### 2.6. Structural Characterizations of Ag NPs Films

UV–vis absorption spectra were measured on a SpectraMax M2 microplate reader (Molecular Devices, Sunnyvale, CA, USA). Transmission electron microscope (TEM) images were acquired using a JEM 2100F microscope (TEM, JEM-2100F, JEOL, Tokyo, Japan). Fourier transform infrared (FTIR) spectroscopy were obtained by a Thermo Antaris II FTIR infrared spectrometer (Thermo Fischer Scientific, Waltham, MA, USA). Raman spectra were obtained using a Raman spectrometer (HORIBA, LabRAM HR Evolution, Horiba, France) at 633 nm. The thermogravimetric analysis (TG) was performed with the (TG SDTA 851e) (Mettler Toledo, Columbus, OH, USA) thermogravimetric analyzer. Changes in color were recorded by a Huawei P30 mobile phone camera. Chilled broiler meat was packaged with a modified atmosphere packaging machine SMART500 (Ulma Packaging, Guipuzkoa, Spain).

### 2.7. Metabolite Extraction

A 200 µL sample was transported to a 1.5 mL Eppendorf microcentrifuge tube. After adding 800 μL of the extract solvent (acetonitrile: methanol, 1:1, containing internal standard: 0.02 mg/mL L-2-chlorophenylalanine), the mixtures were vortexed for 30 s using an XW-80A vortex mixer (Boekel, 270100 Tap Dancer-Vortex Mixer, Feasterville, PA, USA), homogenized at 40 Hz and 5 °C for 30 min using a KQ-300DE ultrasonic extractor (Kunshan Ultrasonic Instruments Manufacture Co., Ltd., Kun shan, China) and rested for 5 min at −20 °C. After 15 min of centrifugation at 13,000 g and 4 °C, supernatant fluid was removed, blown to dryness with nitrogen, and redispersed into 100 μL of an acetonitrile solution (acetonitrile: water = 1:1). The mixtures were vortexed for 30 s and homogenized at 40 Hz and 5 °C for 5 min, followed by centrifugation at 13,000 g and 4 °C for 10 min. The resulting supernatants were transferred to LC–MS vials and stored at −80 °C until the liquid chromatography–mass spectrometry (LC/MS) analysis. All samples were mixed into quality control (QC) samples by taking equal amounts (20 µL) of supernatant.

### 2.8. Liquid Chromatography–Mass Spectrometry Analysis

This method referred to our previous work [29].

## 3. Results and Discussion

### 3.1. Synthesis and Characterization of Ag NP Films

**Figure 1:**
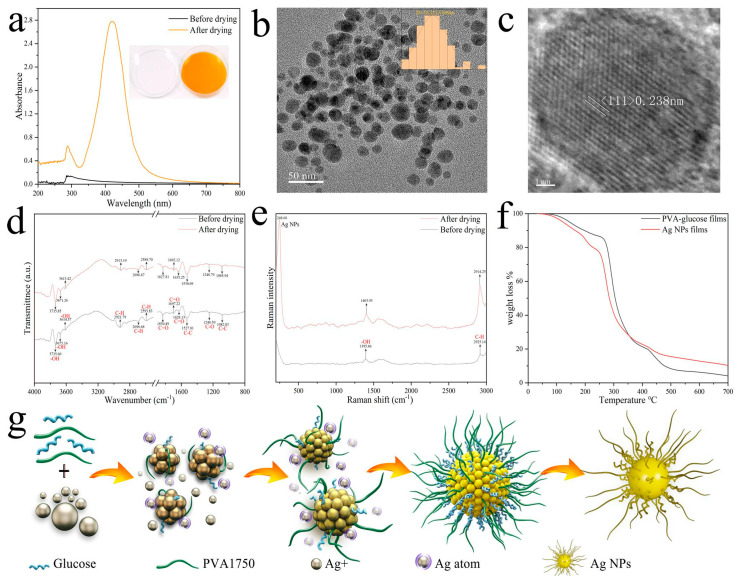
(**a**) Digital photographs of colorless and transparent mixed solution before drying and yellow Ag NP films after drying, and the corresponding UV−visible spectra were recorded; (**b**) particle size and TEM image of Ag NPs; (**c**) HRTEM image of Ag NPs; FTIR (**d**) and Raman (**e**) spectra of colorless and transparent mixed solution before drying and yellow Ag NP films after drying; (**f**) TGA analysis of PVA−glucose films and Ag NP films; (**g**) A mechanism for nanoparticle formation of Ag NPs.

To obtain Ag NP films, the colorless and transparent mixed solution was prepared by mixing PVA, glucose, and AgNO_3_, which were oven-dried for approximately 5 h at 50 °C to evaporate the water, resulting in the formation of yellow Ag NP films, and the corresponding UV−visible spectra showed the characteristic peaks of Ag NPs appearing in the Ag NP film (Figure 1a). Figure 1b shows a TEM image of the Ag NPs, indicating that the uniform dispersion of the Ag NPs is good. The particle sizes (the diameter of the metallic core) were measured and combined into a histogram, and the particle size of these NPs was 16.22 ± 3.89 nm. Measuring the distances of the lattice planes was conducted using high-resolution transmission electron microscopy (HRTEM), as shown in Figure 1c. The crystalline Ag 111 planes with a lattice space of 0.238 nm indicated that the Ag NPs possess a face-centered cubic [FCC] structure, which conforms to the standard lattice constant of the Ag crystal structure. It can be observed that the Ag NPs are nanocrystalline and do not have any amorphous structure [30].

The FT-IR was used to detect the functional groups of mixed solution and Ag NP films. As shown in the Figure 1d, regarding the FTIR spectrum of the mixed solution before drying, twelve functional groups were observed including the O–H group at 3735.60, 3675.16, and 3614.57 cm^−1^ and the C–H group at 2921.79, 2696.68, and 2593.83 cm^−1^. The peaks centered at 1830.49, 1697.22 and 1628.13 cm^−1^ were ascribed to the C=O group, and the C−C and C−O groups were in the regions of 1527.81, 1082.83, and 1248.59 cm^−1^. These main functional groups can bind easily with metallic NPs by chelation and form a Ag NP core complex [31,32,33]. These peaks were also detected in the Ag NP films after drying. However, most of them appeared at lower wavenumbers indicating their active participation in the reduction of Ag ions and thus the formation of Ag NPs [34]. From this, it was concluded that the PVA and glucose were responsible for the reduction, stabilization, and formation of Ag NPs.

Based on our investigations of the Ag NP films detected from the FTIR spectroscopy, we have shown Raman spectra from the same treated samples in Figure 1e. Generally, due to the surface-enhanced Raman spectroscopy (SERS) characteristics of Ag NPs, the adsorption surface of Ag NPs has a noticeable significantly enhanced Raman signal. The characteristic Raman shift peaks of Ag NPs were observed at 248.68 cm^−1^, refs [35,36] which indicated that Ag NPs were synthesized via water evaporation-induced self-assembly methods. The spectra under 633 nm wavelength excitations clearly show strong -OH (1403.93 cm^−1^) and C−H (2914.25 cm^−1^) groups Raman signals of Ag NP films which can be seen in Figure 1e. The signal sharpness and intensity of the characteristic peaks were obviously enhanced indicating that the bonds capped the surface of Ag NPs. The results were consistent with FTIR spectra.

TG analysis of PVA−glucose films and Ag NP films is shown in Figure 1f. PVA–glucose films and Ag NP films were thermally stable up to 100 °C. Beyond the first stage, thermogravimetric analysis showed decomposition in the range of 250−350 °C, which is the thermal decomposition temperature of PVA and glucose. [37] Then, there was also a slight weight-loss stage up to 450 °C that could be related to the decomposition of the lattice structure of both samples, but the PVA−glucose films have a higher degree of weight loss. Therefore, it has to be noticed that the total weight loss of Ag NP films is lower than that of PVA–glucose films because the presence of Ag NPs that increase the thermal stability which is consistent with Peihua Ma et al. [37], who detected the enhanced thermal stability of the meta−organic framework after modifying it with PVA.

The nucleation and growth of Ag NPs can be explained by the model by LaMer [38]: at the beginning of the reaction, Ag ions are reduced to zero-valent Ag atoms by PVA and glucose in solution. As the desiccation time increases, the concentration of zero−valent Ag atoms in the films increases continuously. When the concentration of zero−valent Ag atoms reaches a critical value, the atoms will aggregate with each other in the solution to form clusters, which will then separate from the liquid phase to form a solid-phase nucleus. The newly formed zero−valent Ag atoms will grow on the existing solid-phase nucleus until the final Ag NP solution is generated when the Ag atoms on the surface of the nanocrystals are in equilibrium with the zero−valent Ag atoms in the solution. The PVA and glucose ligands are simultaneously attached as ligand molecules to the surface of the Ag NPs. In this mixed ligand system, PVA and glucose ligands exhibit a sheet-like concentration and regional separation distribution on the surface of the Ag NPs, which significantly increases the stability and solubility of the Ag NPs. At the same time, these ligands are not fixed but reciprocate on the surface of the Ag NPs [39]. The schematic process for the nucleation and growth of Ag NPs is shown in Figure 1g.

### 3.2. Sensing Mechanism

Histamine was selected as the model exogenous substance in this experiment since histamine is the major metabolite in chilled broiler meat juice (meat exudate) of vacuum-packaged chilled broiler meat (Table S1). Histamine content in the chilled broiler meat juice (meat exudate) of a low concentration can be detected according to the experimental results.

**Figure 2:**
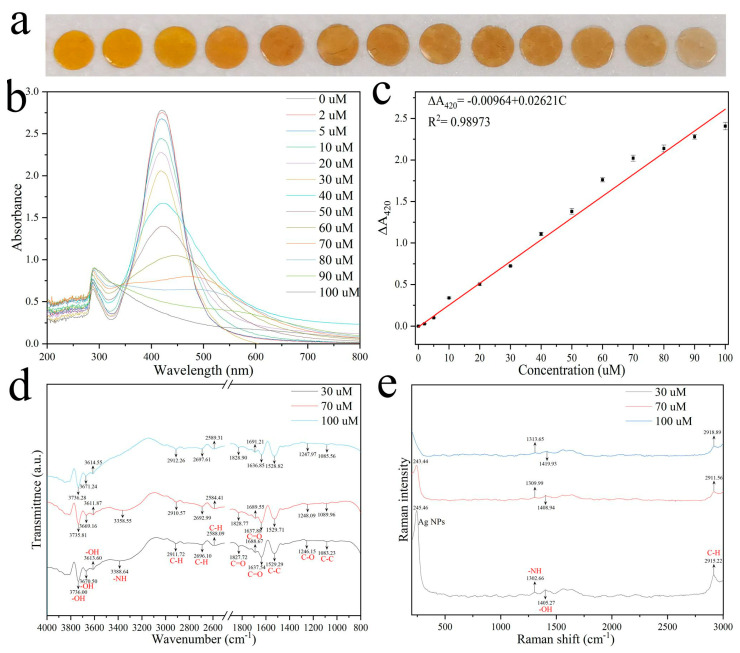
(**a**) Photographs of Ag NP films exposed to histamine at different concentrations. (**b**) UV−vis spectra of Ag NPs films exposed to histamine at different concentrations. (**c**) Relationship between ΔA_420_ and histamine concentrations, the error bars represent the standard deviation for five trials (*n* = 5); FTIR (**d**) and Raman (**e**) spectra of Ag NP films exposed to histamine at different concentrations.

The Ag NP films were cut into 8-millimeter-diameter circular sheets. Then, they were immersed into different histamine solutions (1 mL, 0–100 µM) for 2 min. As shown in Figure 2a, the Ag NP films showed a yellow to pale brown to colorless color change with increasing concentrations of histamine, suggesting their applicability for histamine detection. In order to reveal the sensing mechanism, the UV–vis spectra of Ag NP films exposed to histamine at different concentrations were monitored. As shown in Figure 2b, the LSPR peak for Ag NP films was 420 nm. Ag NP films react with increasing concentrations of histamine. The plasmonic peak originally at ~420 nm shows a red-shift and is gradually weakened and finally disappears, indicating that the Ag NPs were gradually etched by histamine, and finally the Ag particles are completely removed. The results indicate that this sensing relies on the fact that an increase in histamine content results in obvious absorption wavelength intensity change caused by aggregation and etch of the Ag NPs accompanied by obvious color change, thus enabling visual readings and UV–vis quantitation of histamine exposure. The A_420_ of Ag NPs decreased gradually with increasing histamine concentration. ΔA_420_ has a linear relationship with histamine concentration (Figure 2c). The linear regression equation is ΔA_420_ = −0.00964 + 0.02621C (*n* = 5). We also provide the coefficient of determination of the linear regression, R^2^, between the ΔA_420_ and histamine concentration as a measure of the normalization approaches accuracy, with values closer to one indicating better accuracy. The result shows that the ΔA_420_ was highly correlated with H2S concentration (R^2^ = 0.98973).

Changes in functional groups and chemical bonding were determined by FTIR spectroscopy. Notably, we characterized the same Ag NP films exposed to histamine at different concentrations (30, 70, 100 µM) to exclude interference from different batches. For FTIR spectroscopy it could be seen from Figure 2d that the new broad peak appearing at 3388.64 cm^−1^ was the characteristic absorption peak of the NH−stretching of histamine when the histamine concentration was 30 µM, which means the NH−stretching interacts and coordinates with Ag+ ions adsorbed on the surface of Ag NPs, indicating that Ag−NH complexes were formed, which also reflected interactions between H, N, and metal atoms. The NH−stretching peak disappeared as the histamine concentration was increased to 100 µM. These results clearly demonstrate that histamine is involved in the interaction with Ag NPs. The NH−stretching interacts and coordinates with metal atoms adsorbed on the surface of Ag NPs, resulting in the aggregation and etching of the Ag NP particles.

Based on our investigations of the Ag NP films detected from FTIR spectroscopy, we have shown Raman spectra from the same treated samples in Figure 1e. The characteristic Raman shift peaks of Ag NPs gradually decrease in intensity and eventually disappear as the concentration of histamine increased. This provides further evidence that histamines lead to the etching of Ag NPs.

### 3.3. Selectivity Test

**Figure 3:**
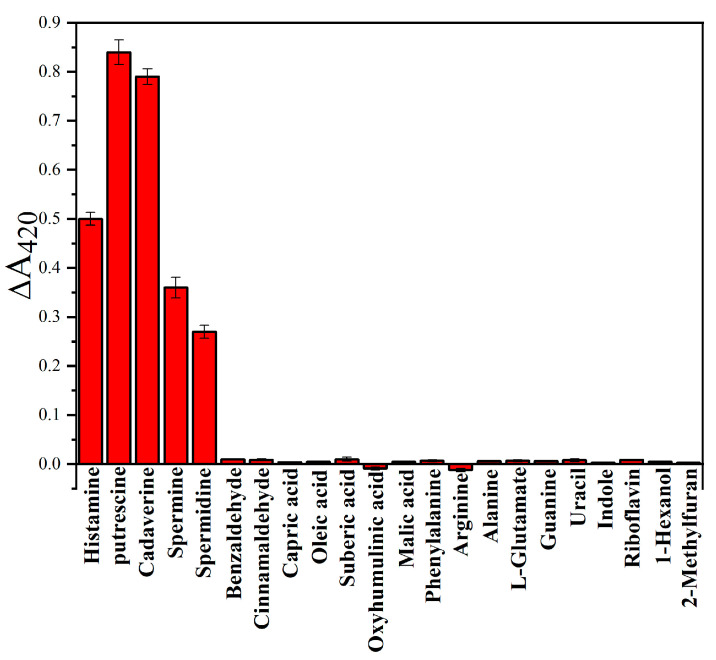
Absorbance of Ag NP films decreased at 420 nm in reaction with BAs or interfering compounds (Histamine, Putrescine, Cadaverine, Spermine, Spermidine: 20 mg/m^3^. Benzaldehyde, Cinnamaldehyde, Capric acid, Oleic acid, Suberic acid, Oxyhumulinic acid, Malic acid, Phenylalanine, Arginine, Alanine, L-Glutamate, Guanine, Uracil, Indole, Riboflavin, 1-Hexanol, 2-Methylfuran: 50 mg/m^3^). Each data point is the mean of six replicate samples (mean ± standard deviation).

LC-MS analysis revealed 49 chilled broiler meat juices (meat exudate) that were present during storage at 4 °C and vacuum-packaged conditions (Table S1), including BAs, aldehydes, acids, amino acids, nucleotides, and others. To evaluate whether our Ag NP films can be used to monitor meat freshness and further to evaluate the sensitivity and selectivity of this sensing method based on Ag NP films in accurately reporting BA levels in actual samples, several representative compounds were evaluated under the same conditions. We tested the responses of Ag NP films to other BAs typically detected in chilled broiler meat, including histamine, putrescine, cadaverine, spermine, and spermidine, which are useful freshness indicators. The Ag NP films were immersed into different BA solutions (20 µM) for 2 min. Since there is a different structure of each amine, the Ag NP films triggered different aggregation and etch processes and further elicit changes in the UV–vis spectra at 420 nm as shown in Figure 3. In contrast, no color change was observed after the introduction of other exudates (50 µM), and all exudates had higher concentrations than BAs. It is particularly worth mentioning that Ag NP films exhibit less response to benzaldehyde, capric acid, phenylalanine, indole, 1-hexanol, and 2-methylfuran. These compounds have strong complexation ability and can etch Ag NP films to interfere with the detection of BAs when Ag NP films are used. These results show that the measured compounds have a negligible effect on the detection of BAs, and the proposed method has high selectivity for BAs. What is more, the Ag NP films showed a similar colorimetric response to other BAs, which provided feasibility for monitoring chilled broiler meat spoilage with total BA content.

**Figure 4:**
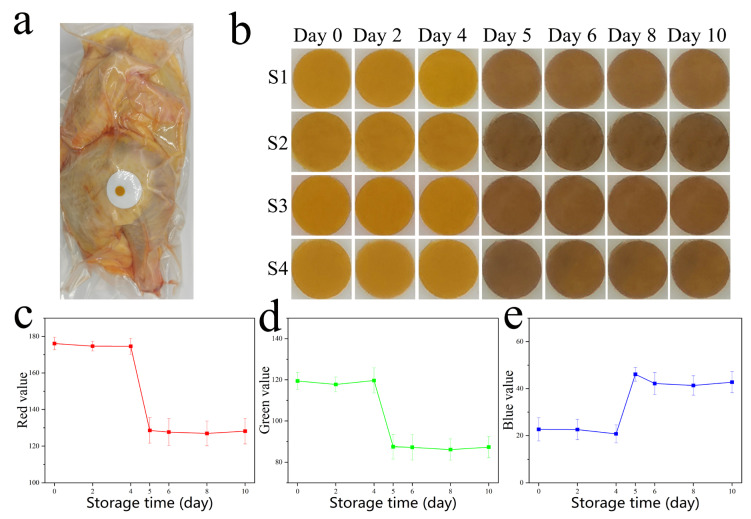
(**a**) The schematic of the chilled broiler meat in vacuum-packaged conditions; (**b**) color change in Ag NPs films in chilled broiler meat in vacuum-packaged conditions at 4 °C, and the assays were repeated four times: S1–S4; (**c**–**e**) Red value, Green value, Blue value of Ag NPs films at 4 °C.

These Ag NP films require meat quality verification with accuracy comparable to existing quality indicators such as TVC and TVB-N content. In order to promote the wide application of the proposed method, Ag NP films were applied to chilled broiler meat under vacuum packaging conditions to monitor the change in the freshness of the meat. The Ag NP films were attached to 2-centimeter-diameter round Tyvek paper, and Tyvek paper keeps the Ag NP films from being in contact with the meat or prevents leaking while still being in contact with the Bas in vacuum-packaged conditions (Figure 4a). The packages were stored at 4 °C, and mobile phone images were taken at fixed times without opening the packaging. As shown in Figure 4b, four chilled broiler meat samples were tested for colorimetric sensing properties. The colorimetric sensing label exhibited color changes from yellow to brown. A smartphone can also be used to obtain digital images in order to obtain the color coordinates, and RGB is one of the most commonly used color models in image processing. RGB value (Red value, Green value, Blue value) models use transmitted light to represent colors and are the most common color model used in digital image color measurement. Thus, we used the value of pixel brightness in an RGB color model to evaluate the color of the Ag NP film image and further analyzed its correlations with quality indicator data. A significant improvement can be shown visually by using an RGB color model to guide the Ag NP film image and by combining the RGB feature image with the Ag NP film image. Digital color images of the Ag NP films were regularly recorded with a Huawei P30 mobile phone camera. Images were imported into Adobe Photoshop 2020 software, and average RGB pixel intensity was collected. All images of the Ag NP films were captured three times, and the final RGB values were determined by the average of each RGB value. When the Ag NP films are used to monitor the spoilage of meat (1000 ± 20 g per sample), the R and G values of the Ag NP films decrease significantly, while the B value increases significantly (Figure 4c–e).

**Figure 5:**
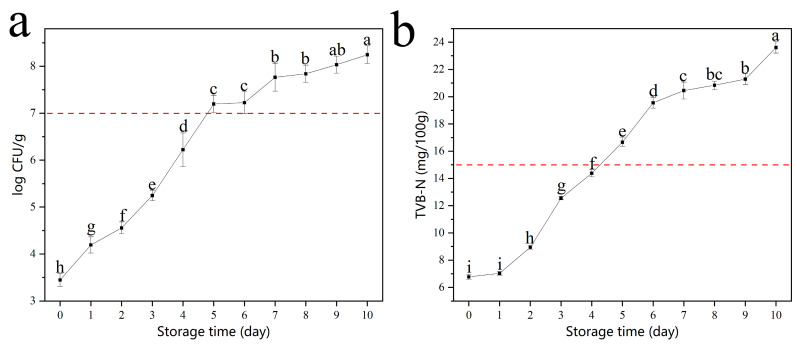
Changes in (**a**) TVCs and (**b**) TVB-N in chilled broiler meat in vacuum-packaged conditions stored at 4 °C. Vertical bars represent standard deviation of the mean (*n* = 6). Different lowercase letters indicate significant differences at different storage times (*p* < 0.05).

Microbiological and physicochemical parameters (such as TVCs and TVB-N) related to meat quality will change over time. According to the International Commission on Microbiological Specifications for Foods (ICMSF), the maximum acceptable level of the TVCs in meat is 7 log CFU g^−1^ [40]. According to the Chinese national standard GB 2707—2016, the safety limit of TVB-N is 15 mg 100 g^−1^. The concentrations of TVCs and TVB-N of chilled broiler meat in vacuum-packaged conditions were monitored. The safety limit of TVCs in chilled broiler meat for human consumption is below 7.0 log CFU g^−1^. As shown in Figure 5a, the TVC levels were 3.45 log CFU g^−1^ and increased with storage time. In addition, TVC levels exceeded the threshold of 7.0 log CFU g^−1^ (7.20 log CFU g^−1^) in about 5 days at 4 °C. The initial value of TVB-N was 6.77 mg 100 g^−1^. The TVB-N level in the meat stored at 4 °C increased with storage time and exceeded 15 mg 100 g^−1^ (16.66 mg 100 g^−1^) on the fifth day of storage (Figure 5b). As shown in Figure 4b, the color of the Ag NPs film was yellow when the meat was very fresh under vacuum packaging conditions at 4 °C, indicating the best flavor of the chilled broiler meat. The color changed from yellow to brown on day 5, which was consistent with the lowest spoilage-level data at 4 °C. The results showed that the prepared Ag NP films had great responsiveness to the changes in the microbiological and physicochemical parameters in the chilled broiler.

## 4. Conclusions

Overall, simple, online, and sensitive detection of meat freshness was of great significance to ensure meat safety and has practical application value in the meat business. We developed a simple method for the preparation of large-area Ag NP films based on evaporation-induced self-assembly. The as-prepared Ag NP films exhibited a linearly dependent response to BAs, including histamine, putrescine, cadaverine, spermine, and spermidine. Upon contacting with histamine, the Ag NP film color immediately changed from yellow to brown to colorless, and the plasmonic peak at 420 nm was linearly proportional to the histamine concentration in the range of 2 µM to 100 µM. The Ag NP films also showed good sensitivity toward other BAs generated from chilled broiler meat during storage, which presented visible color changes when it was used as a BA sensor to monitor chilled broiler meat spoilage. A real-time colorimetric Ag NP film was developed to detect the spoilage of meat using RGB digital images and was compared with the standard food monitoring methods, TVCs and TVB-N. The Ag NP films could be used as a low-cost and portable colorimetric BA sensor for convenient, non-destructive, and instrument-free monitoring of chilled broiler meat spoilage in intelligent packaging.

## Data Availability

Data is contained within the article or supplementary material.

## Supplementary Materials

The following supporting information can be downloaded at: https://www.mdpi.com/article/10.3390/foods12061285/s1, Table S1. The amount of chilled broiler meat juice (meat exudate) during the storage of chilled broiler meat under 4 °C and vacuum-packaged conditions.
