# Supplementary material for: Facile Synthesis of Ag NP Films via Evaporation-Induced Self-Assembly and the BA-Sensing Properties
Source: Foods. 2023 Mar 17;12(6):1285. doi: 10.3390/foods12061285 (PMC10048188; doi:10.3390/foods12061285)
Supplement: Supplementary file 1 [file foods-12-01285-s001.zip › foods-2256103-supplementary.pdf]

Supporting Information

Facile Synthesis of Ag NP Films via Evaporation-Induced Self-Assembly and the BA-Sensing Properties

Jiahang Yu, Huixin Tian, Mingyuan Huang and Xinglian Xu *

Key Laboratory of Meat Processing and Quality Control, Ministry of Education, National Center of Meat Quality and Safety Control, Jiangsu Collaborative Innovation Center of Meat Production and Processing, Quality and Safety Control, College of Food Science and Technology, Nanjing Agricultural University, Nanjing 210095, China

* Correspondence: xlxus@njau.edu.cn; Tel.: +86-025 84395939; Fax: +86-025 84395730

Table S1. The amount of chilled broiler meat juice (meat exudate) during the storage of chilled broiler meat under 4 ℃ and vacuum-packaged conditions. Means within a row followed by different letters are significantly different ($P < 0.05$).

Chilled Broiler Meat Storage at 4 ℃								
Exudate (mg/L)	0 Day	2 Day	4 Day	5 Day	6 Day	7 Day	8 Day	10 Day
Histamine	0.275 ± 0.02^{f}	0.32 ± 0.024^{f}	1.44 ± 0.06^{e}	1.6 ± 0.07^{d}	2.49 ± 0.09^{c}	2.43 ± 0.06^{c}	3.97 ± 0.14^{b}	5.11 ± 0.14^{a}
putrescine	0.02 ± 0.005^{d}	0.04 ± 0.004^{c}	0.039 ± 0.002^{c}	0.041 ± 0.004^{c}	0.05 ± 0.002^{b}	0.08 ± 0.002^{a}	0.079 ± 0.003^{a}	0.08 ± 0.003^{a}
Cadaverine	0	0	0	0.0008 ± 0.001^{d}	0.001 ± 0.001^{d}	0.006 ± 0.003^{c}	0.017 ± 0.005^{b}	0.03 ± 0.003^{a}

Spermine	$0.057±0.013^{e}$	$0.09±0.006^{e}$	$0.217±0.024^{d}$	$0.265±0.025^{d}$	$0.6±0.05^{c}$	$0.71±0.04^{b}$	$0.72±0.03^{b}$	$0.85±0.028^{a}$
Spermidine	$0.392±0.022^{g}$	$0.42±0.045^{g}$	$0.708±0.04^{f}$	$0.798±0.023^{e}$	$0.9±0.03^{d}$	$1.08±0.08^{c}$	$1.16±0.07^{b}$	$1.41±0.025^{a}$
Benzaldehyde	$0.218±0.032^{g}$	$0.37±0.018^{f}$	$0.47±0.04^{e}$	$0.56±0.04^{d}$	$0.77±0.012^{c}$	$0.83±0.03^{b}$	$0.83±0.035^{b}$	$1.03±0.06^{a}$
Phenylacetaldehyde	$0.012±0.002^{g}$	$0.018±0.001^{f}$	$0.027±0.002^{e}$	$0.039±0.003^{d}$	$0.05±0.003^{c}$	$0.05±0.002^{c}$	$0.08±0.002^{b}$	$0.11±0.008^{a}$
Cinnamaldehyde	$0.027±0.001^{a}$	$0.025±0.001^{ab}$	$0.024±0.002^{b}$	$0.018±0.002^{c}$	$0.012±0.001^{d}$	$0.014±0.001^{d}$	$0.014±0.001^{d}$	$0.012±0.001^{d}$
Oleic acid	$1.7±0.04^{a}$	$1.26±0.04^{b}$	$0.26±0.013^{c}$	$0.23±0.008^{c}$	$0.08±0.004^{d}$	$0.06±0.002^{de}$	$0.027±0.002^{e}$	0
Linoleic acid	$0.16±0.005^{a}$	$0.13±0.003^{b}$	$0.095±0.003^{c}$	$0.053±0.002^{d}$	$0.033±0.003^{e}$	$0.03±0.002^{e}$	$0.026±0.001^{f}$	$0.024±0.002^{f}$
Suberic acid	$0.012±0.001^{d}$	$0.014±0.001^{d}$	$0.011±0.001^{d}$	$0.07±0.003^{d}$	$0.103±0.01^{d}$	$0.4±0.03^{c}$	$0.89±0.08^{b}$	$1.59±0.2^{a}$
4-Pentenoic acid	$1.81±0.11^{a}$	$1.7±0.07^{b}$	$1.24±0.04^{c}$	$1.23±0.035^{c}$	$1.08±0.04^{d}$	$0.78±0.06^{e}$	$0.28±0.02^{f}$	$0.24±0.02^{f}$
D-Pantothenic acid	$0.159±0.007^{a}$	$0.1±0.005^{b}$	$0.084±0.003^{c}$	$0.064±0.001^{d}$	$0.066±0.003^{d}$	$0.034±0.002^{e}$	$0.016±0.007^{f}$	$0.013±0.002^{f}$
Arabinonic acid	$14.56±0.14^{h}$	$16.7±0.34^{g}$	$22.087±0.67^{f}$	$23.9±0.6^{e}$	$25.93±0.22^{d}$	$27.68±0.4^{c}$	$34.49±0.75^{a}$	$32.79±1.3^{b}$
Ferulic acid	$0.087±0.003^{b}$	$0.11±0.01^{a}$	$0.063±0.001^{c}$	$0.06±0.002^{c}$	$0.05±0.003^{d}$	$0.033±0.004^{e}$	$0.022±0.002^{f}$	$0.022±0.003^{f}$
Oxyhumulinic acid	$0.014±0.002^{f}$	$0.024±0.002^{d}$	$0.02±0.002^{e}$	$0.02±0.002^{de}$	$0.032±0.004^{c}$	$0.031±0.002^{c}$	$0.042±0.02^{b}$	$0.064±0.002^{a}$
Cucurbic acid	$0.09±0.003^{a}$	$0.098±0.007^{a}$	$0.054±0.004^{c}$	$0.056±0.004^{c}$	$0.073±0.003^{b}$	$0.054±0.002^{c}$	$0.047±0.002^{d}$	$0.058±0.002^{c}$
Capric acid	$0.09±0.012^{d}$	$0.099±0.01^{cd}$	$0.1±0.008^{bc}$	$0.1±0.006^{bcd}$	$0.099±0.004^{cd}$	$0.096±0.004^{cd}$	$0.12±0.007^{a}$	$0.11±0.01^{ab}$
Malic acid	$329.59±28^{c}$	$383.26±20^{a}$	$353±15.4^{b}$	$173.49±8.1^{d}$	$66.43±2.8^{e}$	$42.24±1.66^{f}$	$42.11±3^{f}$	$31.15±2.3^{f}$
Gluconic acid	$70.87±3.9^{a}$	$69.88±2.3^{a}$	$69.3±2.9^{a}$	$62.64±3.2^{b}$	$47.37±2.98^{c}$	$4.09±0.5^{d}$	$4.02±0.26^{d}$	$3.82±0.3^{d}$
Phenylalanine	$13±0.58^{e}$	$21.41±1.45^{d}$	$22.46±2.6^{d}$	$24.52±3.2^{d}$	$33.4±3.1^{c}$	$38.95±2.33^{b}$	$41.42±2.47^{b}$	$59.77±3.6^{a}$
Triacanthine	0	0	0	$0.016±0.001^{e}$	$0.021±0.003^{d}$	$0.051±0.001^{c}$	$0.06±0.002^{a}$	$0.056±0.003^{b}$
Cystathionine	0	0	0	0	0	$0.015±0.003^{b}$	$0.017±0.004^{b}$	$0.021±0.001^{a}$
Arginine	$1.37±0.037^{c}$	$1.49±0.04^{b}$	$1.75±0.03^{a}$	$0.36±0.035^{d}$	$0.31±0.02^{e}$	$0.15±0.007^{f}$	0	0
Lysine	$1.02±0.068^{c}$	$1.95±0.13^{a}$	$1.5±0.04^{b}$	$0.92±0.086^{c}$	$0.73±0.01^{d}$	$0.64±0.003^{d}$	$0.3±0.013^{e}$	$0.284±0.03^{e}$
Tryptophan	$12.2±0.774^{d}$	$13.41±1.6^{d}$	$18.27±0.58^{c}$	$20.74±1.5^{c}$	$42.52±2.5^{b}$	$49.41±3.4^{a}$	$44.4±2.1^{b}$	$51.66±0.22^{a}$
Histidine	$25.2±1.64^{e}$	$27.17±3.4^{de}$	$30.7±1^{cd}$	$34.56±1.72^{c}$	$38.74±1.47^{b}$	$46.8±2.5^{a}$	$48.7±2.9^{a}$	$50.19±4^{a}$
Alanine	0	0	0	0	0	0	$0.01±0.001^{b}$	$0.012±0.002^{a}$
Proline	$0.24±0.03^{a}$	$0.204±0.03^{b}$	$0.16±0.004^{c}$	$0.11±0.016^{d}$	$0.09±0.007^{d}$	$0.062±0.005^{e}$	$0.06±0.003^{e}$	$0.06±0.002^{e}$

Glycine	0.013±0.002^{a}	0.01±0.001^{b}	0	0	0	0	0	0
Methionine	0.029±0.002^{a}	0.023±0.005^{b}	0.013±0.003^{c}	0.01±0.002^{c}	0	0	0	0
Leucine	0.55±0.022^{f}	0.84±0.03^{e}	0.84±0.05^{e}	0.95±0.02^{d}	1.34±0.07^{c}	1.37±0.05^{c}	1.63±0.03^{b}	1.8±0.05^{a}
Tyrosine	0.93±0.024^{a}	0.85±0.04^{b}	0.819±0.04^{b}	0.83±0.047^{b}	0.52±0.01^{c}	0.53±0.03^{c}	0.42±0.02^{d}	0.28±0.01^{e}
L-Glutamate	1.02±0.042^{a}	0.89±0.04^{b}	0.74±0.04^{c}	0.626±0.03^{d}	0.5±0.01^{e}	0.37±0.016^{f}	0.22±0.03^{g}	0.13±0.02^{h}
L-Valine	0.62±0.134^{e}	0.58±0.21^{e}	1.17±0.3^{d}	2.17±0.28^{c}	2.46±0.2^{c}	2.94±0.3^{b}	3.49±0.26^{a}	2.46±0.2^{c}
Hypoxanthine	35.69±1^{f}	39.66±1.56^{e}	42.68±0.8^{d}	45.78±1.26^{c}	47.08±2.2^{c}	60.1±2.26^{b}	63.9±1.6^{a}	61.83±2.2^{ab}
Xanthosine	11.85±0.53^{d}	21.71±1.6^{a}	17.29±0.3^{b}	17.36±0.7^{b}	15.63±0.76^{c}	8.21±0.35^{e}	0.14±0.02^{f}	0.71±0.03^{f}
Adenine	0.07±0.004^{f}	0.11±0.004^{f}	0.2±0.013^{e}	0.26±0.03^{e}	0.35±0.005^{d}	0.58±0.03^{c}	1±0.08^{b}	1.9±0.12^{a}
Guanine	0.019±0.003^{f}	0.032±0.001^{e}	0.034±0.002^{de}	0.048±0.004^{c}	0.07±0.004^{a}	0.063±0.002^{b}	0.038±0.003^{d}	0.03±0.004^{e}
Guanidylic acid	17.85±0.5^{b}	18.89±0.48^{a}	12.72±0.96^{c}	4.91±0.47^{e}	6.14±0.26^{d}	0.21±0.01^{f}	0.11±0.05^{f}	0.15±0.02^{f}
Uracil	0.136±0.018^{c}	0.166±0.005^{b}	0.14±0.004^{c}	0.17±0.01^{b}	0.2±0.018^{a}	0.2±0.008^{a}	0.2±0.01^{a}	0.21±0.01^{a}
Inosine	0.838±0.027^{b}	1.23±0.06^{a}	0.48±0.02^{cd}	0.51±0.01^{c}	0.45±0.016^{d}	0	0	0
Indole	0.57±0.16^{e}	1.81±0.3^{cd}	1.65±0.067^{d}	1.68±0.3^{d}	2.12±0.1^{bc}	2.23±0.13^{b}	2.45±0.36^{b}	4.29±0.23^{a}
Carnosine	0.24±0.03^{bc}	0.25±0.008^{b}	0.22±0.02b^{c}	0.21±0.03^{c}	0.26±0.02^{b}	0.24±0.006^{bc}	0.23±0.02^{bc}	0.38±0.03^{a}
Mytilin A	5.98±0.3^{a}	5.09±0.3^{b}	3.27±0.22^{d}	4.26±0.28^{c}	4.05±0.3^{c}	3.87±0.24^{c}	2.76±0.28^{e}	1.61±0.1^{f}
Riboflavin (Vitamin B2)	1.29±0.06^{c}	2.66±0.16^{a}	1.53±0.03^{c}	1.52±0.03^{c}	2.31±0.18^{b}	1.28±0.26^{c}	1.42±0.13^{c}	2.45±0.2^{ab}
Glutathione	0.44±0.02^{b}	0.43±0.03^{b}	0.31±0.023^{d}	0.4±0.02^{bc}	0.38±0.03^{c}	0.59±0.03^{a}	0.14±0.03^{e}	0.11±0.03^{e}
1-Hexanol	0.12±0.002^{e}	0.17±0.001^{d}	0.21±0.006^{bc}	0.23±0.01^{b}	0.3±0.03^{a}	0.22±0.009^{bc}	0.2±0.01^{c}	0.17±0.006^{d}
2-Methylfuran	0.011±0.001^{c}	0.012±0.001^{c}	0.011±0.001^{c}	0.012±0.001^{c}	0.013±0.001^{bc}	0.012±0.001^{c}	0.016±0.002^{b}	0.02±0.004^{a}
